# Association of *IGF-I* gene polymorphisms with milk yield and body size in Chinese dairy goats

**DOI:** 10.1590/S1415-47572010005000034

**Published:** 2010-06-01

**Authors:** Chanjuan Deng, Rongnuan Ma, Xiangpeng Yue, Xianyong Lan, Hong Chen, Chuzhao Lei

**Affiliations:** 1 Shaanxi Key Laboratory of Molecular Biology for Agriculture, College of Animal Science and Technology, Northwest AF University, Yangling, ShaanxiChina; 2 Institute of Cellular and Molecular Biology, Xuzhou Normal University, Xuzhou, JiangsuChina

**Keywords:** *IGF-I gene*, dairy goat, milk yield, PCR- SSCP

## Abstract

The association of *IGF-I* gene polymorphisms with certain traits in 708 individuals of two Chinese dairy-goat breeds (Guanzhong and Xinong Saanen) was investigated. Polymerase chain reaction-single strand conformation polymorphism (PCR-SSCP) and DNA sequencing methods were employed in screening for genetic variation. Two novel mutations were detected in the 5'-flanking region and in intron 4 of *IGF-I* gene, viz., g.1617 G > A and g.5752 G > C (accession D26119.2), respectively. The associations of the g.1617 G > A mutation with milk yield and the body size were not significant (p > 0.05). However, in the case of g.5752 G > C, Xinong Saanen dairy goats with the CG genotype presented longer bodies (p < 0.05). Chest circumference (p < 0.05) was larger in Guanzhong goats with the GG genotype. In Xinong Saanen dairy goats with the CC genotype, milk yields were significantly higher during the first and second lactations (p < 0.05). Hence, the g.5752 G > C mutation could facilitate association analysis and serve as a genetic marker for Chinese dairy-goat breeding and genetics.

Animal growth is controlled by a complex system, in which the somatotropic axis plays a key role. Genes that operate in this axis are responsible for postnatal growth, mainly by way of growth hormones acting on bones and muscles mediated by insulin-like growth factor I (*IGF-I*) (Sellier, 2000). Thus, *IGF-I* is an important growth factor involved in a variety of physiological processes including cell differentiation, embryogenesis, and regulation of metabolism, reproduction, fetal development, and growth (Adam *et al.*, 2000; Shen *et al.*, 2003). The *IGF-I* gene was mapped on chromosome 5 in bovine (Miller *et al.*, 1991). The *IGF-I* gene is expressed in most tissues, but mainly in liver (Wang *et al.*, 2003). It encodes a polypeptide of molecular weight of 7.5 kDa with 70 amino acids (Daughaday and Rotwein, 1989). The amino acid sequence of *IGF-I* is identical in human, cattle, dog, and pig (Nixon *et al.*, 1999).

In the nineties, several lines of evidence revealed an association of the high level of *IGF-I* in the blood with fast growth in cattle (Barash *et al.*, 1998; Sirotkin *et al.*, 2000). In the following decades, cattle *IGF-I* gene mutations (C/T mutation in the 5'-flanking region) were found to be associated with the percentage of fat and protein in milk (Ge *et al.*, 2001; Eulalia *et al.*, 2006). However, these results were quite different from Hines' discovery whence no association was found between C/T mutation in 5'-flanking region and dairy production traits in Holstein cattle (Hines *et al.*, 1998). The association of *IGF-I* gene polymorphism (short random repeat in the 5'-flanking region) with body weight, both at birth and weaning, has also been described (Li *et al.*, 2004; Moody *et al.*, 1996), although such an association was not found by Curi *et al.* (2005). Recently, an *IGF-I* intron 2 polymorphism was found to correlate positively with the twinning rate (Kim *et al.*, 2009).

Dairy goats are numerous in northwest China, among which, the Xinong Saanen (SN) and Guanzhong (GZ) breeds are the best-known, due not only to their tolerance of crushed feed and the local harsh weather, but also to their high milk yield. As it is difficult to culture high quality domestic breeds over a short period by the traditional breeding and genetics methods, the major breeders recently focused on using DNA markers for developing breeds through marker-assisted selection (MAS). Thus, it is important to identify significant associations between polymorphisms within crucial candidate genes and production traits. The aim was to find *IGF-I* gene SNPs in two Chinese dairy goat breeds and search for the possible associations of these with milk yield and body size.

708 blood samples were collected from Shaanxi province (440 for GZ breed from Sanyuan Guanzhong dairy goat breeding base in Shaanxi province; 202 for SN breed from Qianyang Saanen dairy goat breeding base in Shaanxi province; 66 for SN breed, also from animal farm in Northwest A&F University in Shaanxi province). All goats were 2 to 3 years old adults. Measurements were taken for all the individuals (BH = body height; BL = body length; ChC = chest circumference). Based on a 305-day lactation period, the first and second lactation data from each of 202 SN dairy goats from the Qianyang Saanen dairy goat breeding base, were recorded for every goat.

Blood samples were drawn from the jugular vein into curettes containing ACD (anticoagulant citrate dextrose). The samples were stored at -80 °C for several weeks, whereupon genomic DNA was extracted with the use of phenol/chloroform (Sambrock *et al.*, 2001).

Based on the goat *IGF-I* gene sequence published in the GenBank (D26119.2), five pairs of primers were designed. Primer sequences, annealing temperatures and amplicons are listed in Table 1. The P5 primers for the 5' -flanking end in Table 1 referred to those used in cattle (Ge *et al.*, 2001), whereas the remainder were designed in this study.

The final volume of 25 μL contained 50 ng of genomic DNA, 0.5 μL of each primer (10 pM), 2.5 μL of a 10xbuffer (including 20 mM Mg^2+^), 0.5 μL of dNTPs (10 mM) and 0.25 μL of *Taq* DNA polymerase (5 U/μL Dongsheng. Guangzhou, China). Amplification was carried out using PCR, under the following conditions: 5 min at 96 °C for initial DNA denaturation, 35 cycles of denaturation at 94 °C for 40 s; annealing at X °C (shown in Table 1) for 40 s for each pair of primer; extension at 72 °C for 40 s. The final cycle was followed by a 10 min extension at 72 °C.

Aliquots of 4 μL of PCR products were mixed with 8 μL of denaturing solution (95% formamide, 25 mM EDTA, 0.025% xylene-cyanole and 0.025% bromophenol blue) and heated for 10 min at 98 °C and chilled on ice. Denatured DNA was subjected to 10% PAGE (80 mm x 73 mm x 0.75 mm) in 1x TBE buffer and constant voltage 250V for 15 min, then 160V for 2 h. The gel was stained with 0.1% silver nitrate (Lan *et al.*, 2007). After the polymorphisms were detected, the PCR products of different electrophoresis patterns were sequenced by the Huada Genetic Sequence Company (Beijing, China). The sequences were analyzed with DNASTAR software (Version 7.10, DNASTAR, Madison, USA).

Based on the individual number of different genotypes in analyzed breeds, Hardy-Weinberg equilibriums, genotypic frequencies and allelic frequencies were directly calculated. The statistical software SPSS (Version 16.0) was used to analyze the relationships between the genotypes and traits in goats. The adjusted Linear Model I with fixed effects was used to evaluate the relationships between genotypes and milk yield of Xinong Saanen dairy goats. The model applied was: *Y*_*ijk*_ = μ + *A*_*i*_ + *G*_*j*_ + (*AG*)_*ij*_ + *e*_*ijk*_, where *Y*_*ijk*_ was the trait measured in each *ijk*th animal, μ the overall population mean, *A*_*i*_ the fixed effect due to the *i*th age, *G*_*j*_ the fixed effect associated with the *j*th genotype, *(AG)*_*ij*_ the interaction between the *i*th age and the *j*th genotype, and *e*_*ijk*_ the random error. Effects associated with farm, sex and season of birth (spring versus fall), and the ages of dams and sires, were not considered in the linear model, since preliminary statistical analysis indicated that these effects did not have significant influence on the variability of traits in SN female dairy goats. The adjusted Linear Model II with fixed effects was used to analyze the relationships between genotypes and body size in these two breeds. The model applied was: *Y*_*ijk*_ = μ + *B*_*i*_ + *G*_*j*_ + (*BG*)_*ij*_ + *e*_*ijk*_, where *Y*_*ijk*_ was the trait measured in each *ijk*th animal, μ the overall population mean, *B*_*i*_ the fixed effect due to the *i*th breed, *G*_*j*_ the fixed effect associated with *j*th genotype, *(BG)*_*ij*_ the interaction between the *i*th breed and the *j*th genotype, and finally *e*_*ijk*_ the random error. Effects associated with farm, sex and season of birth (spring versus fall), as well as the ages of both dams and sires, were not used in the linear model, as the preliminary statistical analyses indicated that these effects did not have significant influences on variability of traits in this two breeds. All the goats were adults, so the age did not influence on the body size. The least square means (LSMs), with standard errors and multiple range tests for *IGF-I* genotypes and traits, were calculated.

Using PCR-SSCP method with five pairs of primers of the goat *IGF-I* gene, only two (P1 and P4) of them demonstrated polymorphic patterns in both breeds. In the P1 locus (fragment amplified by the P1 pair of primers), there were only two kinds of SSCP polymorphic patterns detected in all the 708 samples. We named them GG, with three bands, and AG, with five bands (Figure 1a). In the P4 locus (fragment amplified by the P4 pair of primers), three SSCP polymorphic patterns were found in the 708 samples, which were named GG pattern, with four bands, CG pattern, with five bands, and CC pattern also with four bands (Figure 1b).

In order to clarify the nucleotide mutations in these two loci, nine PCR products, each with a different pattern, were sequenced at each locus. The three remaining products (P2, P3, and P5) were also sequenced. On comparing with GenBank sequence D26119.2, 249 bp PCR products, including the 5'-flanking region upstream from the start codon (ATG), were amplified by the PI pair of primers. There was a G to A mutation, namely g.1617 G > A (accession D26119.2). The P4 primers were used to amplify the whole of exon 4 and part of introns 3 and 4. There was a G to C variation in both breeds, viz., g.5752 G > C (accession D26119.2), located in intron 4. No mutation was detected in the remaining loci (P2, P3, and P5).

The allelic and genotypic frequencies of each locus obtained for the different genetic groups, can be seen in Table 2. For the g.1617 G > A mutation (accession D26119.2), frequencies of the *IGF-I-A* allele in the analyzed populations were 0.08 and 0.11 for GZ and SN dairy goats, respectively, with genotype distribution of both breeds in agreement with Hardy-Weinberg equilibrium (p > 0.05). For the g.5752 G > C mutation (accession D26119.2), the GZ breed was not in Hardy-Weinberg equilibrium (p < 0.01), whereas the SN breed was in Hardy-Weinberg equilibrium (p > 0.05). The allelic frequencies for C were 0.19 and 0.30 for GZ and SN dairy goats, respectively (Table 2).

Combined data related to body-size and milk-yield at the first and second lactations are shown in Table 3. For g.1617 G > A (accession D26119.2), there was no significant association with body size and the milk yield in the two breeds (p > 0.05). For g.5752 G > C (accession D26119.2), significant statistical results were encountered in milk yield and BL (body length) of individuals with the CC and CG genotypes (p < 0.05 and p < 0.05, respectively) in Saanen dairy goats. The BL of SN breed with CG genotype was significantly longer than in the others. Individuals with genotype CC of the *IGF-I* gene had a superior milk yield at the first and second location when compared to those of individuals with genotype CG and GG (p < 0.05). In the Guanzhong population, chest circumference (ChC) of GZ goats with the GG genotype was the largest of the three genotypes, whereas ChC for individuals with CG genotype was larger than that for CC carriers (p < 0.05).

When focusing on the *IGF-I* gene, an attempt was made to detect nucleotide mutations in primer amplified fragments, and to analyze their associations with body size and milk yield in Chinese dairy goats. The analyzed length of the *IGF-I* gene was 1588 bp, including part of the 5'- flanking, and almost the entire coding region except for exon 2 which is only 15 bp long. In the g.1617 G > A mutation (accession D26119.2), the lack of AA homozygotes indicated that the mutation in the goat *IGF-I* gene might give rise to diminished production in the population under analysis. These two dairy goat breeds are major milk producers, originating from a varied dairy-goat breeding basis in Shaanxi province (P.R. China). As the two populations were over one year old, individuals with low production had possibly already been eliminated. Hence, it is reasonable to surmise that AA homozygotes is almost completely lost in quite large local populations. Obviously, the reason of the lack of genotype AA in these breeding farms requires further investigation.

Significant statistical results were found as regards body size and milk yield among different genotypes in g.5752 G > C (accession D26119.2) (p < 0.05) (Table 3). Being located in one of the intron regions, the SNP studied here may not be a causal mutation. Thus, we suggest that it could be linked to another mutation in the coding or regulatory regions of a gene which is a causal mutation for production traits. Introns have been shown to affect the transcriptional efficiency of numerous genes in a variety of organisms (Greenwood and Kelsoe, 2003; LeHir *et al.*, 2003). Further analysis is required to validate both the association found in this study and the physiological significance of the intron 4 mutation in *IGF-I* gene.

In conclusion, our study revealed the first known significant associations of *IGF-I* gene polymorphisms with milk yield and body size in dairy goats. The CC genotype at the g.5752 G > C (accession D26119.2) locus could be used as a molecular marker for superior milk yield. Furthermore, this study will be of practical use for the improvement of Chinese native dairy goats and the local breeding of those with higher milk production.

**Figure 1:**
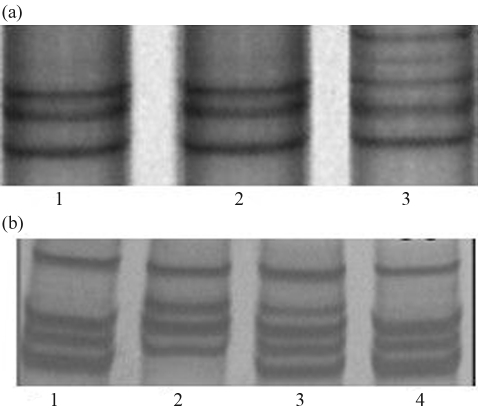
(a) Different PCR-SSCP patterns in the 10% PAGW in P1 locus. Lane 3 was AG pattern; lane 1, 2 were GG pattern. (b) Different PCR-SSCP patterns in the 10% PAGE in P4 locus. Lane 1, 4 were GG pattern; lane 2 was CC pattern; lane 3 was CG pattern.

## Figures and Tables

**Table 1:** PCR primer sequence information and annealing temperatures.

Gene loci	Primer sequences	Annealing temperatures (X °C)	Locations^a^
P1	F: 5'- ATTACAAAGCTGCCTGCCCC -3' R:5'-ACCTTACCCGTATGAAAGGAATATACGT-3'	60.3	1407-1655 (249 bp)
P2	F: 5'- TTCTCTAAATCCCTCTTCTGTTTG -3' R: 5'- CCAATGACTTCAAAGAGTAAG -3'	54.7	1942-2231 (290 bp)
P3	F: 5'- ACCCAGGAGGAAGATGACC -3' R: 5'- ATCCACAGAGCAGCGAGA -3'	65.7	4728-4956 (229 bp)
P4	F: 5'- GCTGGGTGTAGCAGTGAACA -3' R: 5'- GTTGCTTCAGAAGCATAACT -3'	60	5471-5790 (320 bp)
P5	F: 5'- GATAGGTTGGATACATAAA -3' R: 5'- GTTGCGTAGAAAGAAGTG -3'	55.8	6030-6580 (550 bp)

^a^Based on GenBank accession: D26119.2.

**Table 2:** Allelic and genotypic frequencies of P4 and P1 in Chinese dairy goats.

Gene loci	Breeds	Genotypes					Genotypic frequencies				Allelic frequencies			Hardy-Weinberg equilibrium (χ ^2^ test)
		GG	AG	AA	N		P_GG_	P_AG_	P_AA_		P_A_	P_G_		
P1	GZ	373	67	0	440		0.85	0.15	0		0.08	0.92		2.98
	SN	211	57	0	268		0.79	0.21	0		0.11	0.89		3.79

		CC	CG	GG	N		P_CC_	P_CG_	P_GG_		P_C_	P_G_		
P4	GZ	47	69	324	440		0.10	0.16	0.74		0.19	0.81		101.56 (p < 0.01)
	SN	22	116	130	268		0.08	0.43	0.49		0.30	0.70		0.30

χ ^2^_0.05_ (df = 1) = 3.84, χ ^2^_0.01_ (df = 1) = 6.63.

**Table 3:** Relationship between genotypes and body size and milk yield in Chinese dairy goats.

Gene loci	Breeds	Genotypes	Body size (mean ± SE)				Milk yield (mean ± SE)	
			BH(cm)	BL(cm)	ChC(cm)		First lactation (kg)	Second lactation (kg)
	GZ	GG	67.92 ± 0.20	77.62 ± 0.26	92.38 ± 2.39		No record	No record
		AG	67.50 ± 0.47	76.77 ± 0.63	89.82 ± 5.67		No record	No record
P1	SN	GG	74.34 ± 0.24	82.90 ± 0.28	92.56 ± 0.37		627.218 ± 7.661	855.868 ± 10.404
		AG	74.91 ± 0.46	82.67 ± 0.54	93.22 ± 0.71		621.858 ± 15.017	841.138 ± 19.798

	GZ	CC	67.04 ± 0.57	76.94 ± 0.77	87.45 ± 0.89^b^		No record	No record
		CG	67.10 ± 0.47	76.74 ± 0.63	89.29 ± 0.73^ab^		No record	No record
		GG	67.89 ± 0.21	77.65 ± 0.29	90.22 ± 0.33^a^		No record	No record
P4	SN	CC	74.57 ± 0.78	81.47 ± 0.89^b^	91.10 ± 1.14		679.307 ± 23.718^a^	940.317 ± 42.748^a^
		CG	74.57 ± 0.32	83.46 ± 0.37^a^	93.04 ± 0.48		622.313 ± 7.965^b^	858.094 ± 11.856^ab^
		GG	74.05 ± 0.31	81.95 ± 0.35^b^	92.25 ± 0.45		616.236 ± 7.525^b^	833.978 ± 14.808^b^

LSM in a column with no common superscripts differs significantly; low-case character represents significance at p < 0.05.
